# Risk Factors Associated With Increased Anxiety Sensitivity in Children and Adolescents in Northwest China During COVID-19 Pandemic Lockdown

**DOI:** 10.3389/fpsyg.2022.933207

**Published:** 2022-07-08

**Authors:** Qiaoyan Jin, Wenxian Ma, Yang Zhang, Huiyuan Wang, Juanjuan Hao, Yan Geng, Bo Zhong, Jing Li, Wei Hou, Shemin Lu

**Affiliations:** ^1^Department of Pediatrics, The Second Affiliated Hospital of Xi'an Jiaotong University, Xi'an, China; ^2^Department of Biochemistry and Molecular Biology, Xi'an Jiaotong University Health Science Center, Xi'an, China

**Keywords:** anxiety sensitivity, children and adolescents, child psychology, COVID-19 pandemic, lockdowns, quarantine, risk factors

## Abstract

**Purpose:**

A large body of evidence has revealed that the sudden outbreak of public health emergencies induces dramatic effects on the mental health of the general public. We aimed to investigate the level of anxiety sensitivity and its risk factors in children and adolescents from northwest China during the COVID-19 pandemic lockdown in early 2020.

**Methods:**

A cross-sectional survey was conducted through the Wenjuanxing platform using a convenience sampling method between 18 and 26 February 2020. The self-designed questionnaire contained sociodemographic characteristics, factors associated with the COVID-19 pandemic, and the Childhood Anxiety Sensitivity Index (CASI) scale. The data from 1,091 valid questionnaires from students aged 9–17 years were analyzed using ANOVA, multiple linear regression, and binary logistic regression.

**Results:**

The average CASI scores were 11.47 ± 6.631, and 642 students (58.9%) had prominent anxiety sensitivity. Gender, education level, family members participating in anti-COVID-19 work, getting ill and needing medical help during the lockdown, feeling afraid or having heart palpitations on hearing things associated with COVID-19, believing that COVID-19 would have adverse impacts on themselves or their family in the future, and fear of infection were identified as significant factors for elevated levels of anxiety sensitivity (*p* < 0.05). We established a multiple linear regression model for the anxiety sensitivity score. Risk factors found for anxiety sensitivity in children and adolescents during the COVID-19 lockdown included studying in secondary or high school, becoming ill during the pandemic, feeling afraid or experiencing rapid heartbeat or palpitations on hearing about the COVID-19 pandemic, thinking that COVID-19 would have an adverse impact on themselves or their family in the future, and fear of infection.

**Conclusions:**

During the COVID-19 pandemic and home quarantine, scores measuring the prevalence of anxiety sensitivity in children and adolescents from northwest China were elevated. We should develop measures that especially target possible risk factors to intervene against and prevent anxiety sensitivity in children and adolescents in both the current and future pandemics.

## Introduction

Coronavirus disease 2019 (COVID-19) is an emerging infectious disease caused by severe acute respiratory syndrome coronavirus-2 (SARS-CoV-2) (Commission, 2020) that has resulted in a global pandemic with an enormous impact on the health and routine activities of people worldwide (Fang et al., 2021a; Who, 2022). Sufficient data from previous studies on epidemics have revealed that public health emergencies arouse a series of mental health problems besides physical disease. In an investigation of a severe Legionnaires' Disease outbreak in Japan, 13.7% of the survivors demonstrated that they suffered from depressive symptoms (Tsuruta et al., 2005). In a survey on the 2005 SARS pandemic in China, Wu et al. reported that nearly 18% of respondents reported symptoms related to post-traumatic stress disorder (PTSD), anxiety, and depression (Wu et al., 2005). A more recent study reported that 83.1% of respondents had some anxiety about the swine flu outbreak in 2011 (Kanadiya and Sallar, 2011).

COVID-19, without exception, has been found to be a tremendous stressor affecting people's psychological wellbeing. For instance, Tian et al. (2020) found that COVID-19 had significant adverse sociopsychological effects on the Chinese public. Li et al. (2020) found that negative emotions (e.g., anxiety, depression, and indignation) and sensitivity to social risks increased, while scores of positive emotions (e.g., measured by the Oxford Happiness Questionnaire) and life satisfaction decreased 1 week after the declaration of the COVID-19 lockdown. Kim et al. (2021) found a high prevalence of depression and anxiety in society during the COVID-19 pandemic in Korea. Moreover, many Americans have increasingly used prescription drugs to deal with stress and anxiety related to the pandemic (Digon, 2020). These studies consistently showed that people's psychological wellbeing has been negatively influenced by the COVID-19 pandemic and its related control measures. These abnormal conditions of physiology drive behaviors that can include evacuation panic, resistance to public health measures, overburdening of hospitals and clinicians, blaming the government, and abandoning responsibilities to families and jobs. This cascade of effects has caused more severe and disabling ramifications from the COVID-19 pandemic than the disease itself.

Many studies of the impact of the pandemic on psychological conditions have focused on such changes in different populations, such as health care workers (Hao et al., 2021), medical students (ElHawary et al., 2021; Halperin et al., 2021), university students (Jiang, 2020; Mao et al., 2021), confirmed patients (Klaser et al., 2021; Shah et al., 2021), and sick persons with different illnesses (Colombo et al., 2020; Kotecha, 2020; Di Riso et al., 2021). Children and adolescents are a worthwhile study segment, as their comprehension of pandemic-related knowledge is limited, they have insufficient self-protective skills, and they heavily depend on adults for emotional support and physical care (Mollborn and Lawrence, 2018; Leach et al., 2021; Qiu et al., 2021). Therefore, they have been identified as a vulnerable segment of the population in psychosocial characteristics (Perrin et al., 2009; Stevenson et al., 2009) and are prone to suffering mental health problems when coping with disasters (Furr et al., 2010; Pfefferbaum et al., 2015).

Before the COVID-19 pandemic, however, few data existed on the effects of public health emergencies on the mental health of children and adolescents. Many researchers, realizing this lack, made calls for policymakers and clinicians to take the mental health needs of children and adolescents into account when making decisions during the influenza pandemic of 2009 (Perrin et al., 2009; Stevenson et al., 2009). During the COVID-19 pandemic, the psychological conditions of children and adolescents have attracted the attention of researchers. Studies revealed that this demographic suffers from a range of psychological disorders (Wang et al., 2020; Zhou et al., 2020a; McArthur et al., 2021; Ravens-Sieberer et al., 2021), such as depression, anxiety, insomnia, and stress. It is worth noting that, because of varying sociocultural and economic contexts (Dowd et al., 2011; Burgard et al., 2013; Fang et al., 2021b; Wu et al., 2022a), the mental health status of children and adolescents varies by region (Compton et al., 2006; Chen et al., 2020). Studies in China during the COVID-19 pandemic, however, have been conducted on a population mainly from the provinces in southeast and central China, including Henan (Xu et al., 2021), Hubei (Xie et al., 2020), Guangdong (Qin et al., 2021), Guangxi (Chi et al., 2021), Shanghai (Tang et al., 2021), Beijing, and Zhejiang (Chen et al., 2020). Few studies involved children and adolescents from northwest China, where population density is low, the economic situation is poor, and ethnic minorities are concentrated. Therefore, assessing the mental health condition of these children and adolescents was an unmet need.

Anxiety sensitivity refers to the belief that anxiety-related sensory arousal will have negative consequences for the individual, such as death, mental disorders, and social rejection; this belief, in turn, generates fear in the form of primary sensory arousal (Reiss et al., 1986; Taylor et al., 2007). In this way, anxiety sensitivity is a relatively stable indicator that reflects the degree of fear in individuals (Taylor et al., 2007). In addition to being a risk factor for anxiety disorders, anxiety sensitivity can predict anxiety, and non-anxiety disorders (Olatunji and Wolitzky-Taylor, 2009), such as depression, substance abuse, and suicide (Naragon-Gainey, 2010; Oglesby et al., 2015). Therefore, anxiety sensitivity can be used to screen high-risk populations with mental disorders (Schmidt et al., 2010; Noël and Francis, 2011). Indeed, research from the COVID-19 pandemic shows that anxiety sensitivity is increased in adults and is positively associated with suicidal ideation (Allan et al., 2021), depression, and anxiety (Avidor et al., 2021). Taken together, these studies demonstrate that anxiety sensitivity could be a significant predictor of COVID-19-related fear and consequent safety behaviors (Mayorga et al., 2022). Thus, the primary aim of the present study was to assess the anxiety sensitivity of children and adolescents in northwest China early in the pandemic. The purpose was to learn how to take measures to prevent and reduce adverse mental health outcomes and maladaptive behavioral responses resulting from current and future pandemics.

Since the outbreak of COVID-19, many efforts have been made to explore factors influencing psychological abnormalities in children and adolescents (Zhou et al., 2020b; Qin et al., 2021; Ravens-Sieberer et al., 2021; Tang et al., 2021). These researchers have confirmed that risk factors include mainly disturbance of routine life, lack of face-to-face contact with peers, fears of infection, and poor efficiency of online learning. However, the influence of physical conditions on mental health in this period has not been investigated, while evidence has shown that physical disease is a significant influencing factor (Ohrnberger et al., 2017; Felix et al., 2020). To fill these gaps, we explored risk factors associated with anxiety sensitivity in children and adolescents from northwest China during the COVID-19 pandemic. Our purpose was to provide a scientific basis for formulating precise psychological preventions and interventions.

## Methods

It is well-known that cross-sectional questionnaire surveys are generally quick, easy, convenient, and cost-effective to perform. They are particularly suitable for estimating the prevalence of disease in a population and exploring or screening for possible risk factors (Sedgwick, 2014). Thus, we employed a cross-sectional questionnaire in our study. Because of home quarantine during the pandemic, face-to-face interviews could not be conducted. Therefore, the study questionnaire was distributed and retrieved online using the program “Questionnaire Star” (https://www.wjx.cn/), which is widely used and well-recognized as a professional online survey tool (Qin et al., 2021; Tang et al., 2021).

### Study Population and Procedures

Children and adolescents aged 9–17 years were recruited using a convenience sampling method from 18 to 26 February 2020, following a month of the COVID-19 outbreak and subsequent lockdown in China, i.e., the peak of the pandemic. The subjects were mainly from Gansu, Shaanxi, and Xinjiang provinces, all located in northwest China. Teachers sent the link address of the questionnaire to a WeChat group that included teachers, participants, and parents. Then, participants were directed to the Questionnaire Star program by the link address and completed the questionnaire if they were interested. Before filling out the questionnaire, participants provided informed consent. Meanwhile, the teachers were responsible for explaining the manual procedures for the survey in detail. A phone number and WeChat ID for a pediatrician were also included in the questionnaire so that participants could consult and interact with the pediatrician at any time. The entire survey was carried out using voluntary, anonymous, and confidential principles.

### Ethics Statements

In the preface, the purpose and organization of the survey were described. According to the wishes of the students and their guardians, every student filled out the questionnaire voluntarily. All participants could submit, terminate, and repost the questionnaire directly, even though they had started to fill it out with prior consent. The study was approved by the ethics committee of the Second Affiliated Hospital of Xi'an Jiaotong University and carried out following American Association for Public Opinion Research (AAPOR) reporting guidelines.

### Measurements

The survey questionnaire was self-designed and consisted of three sections. Its rationality and functionality were assessed by a pilot study that preceded the study.

#### Sociodemographic Characteristics

Sociodemographic characteristics were chosen by the authors. They included a set of questions regarding sex, age, ethnicity, place of residence, education level, and the number of people residing in the same home during the lockdown.

#### Factors Associated With the COVID-19 Pandemic

Using factors that may affect anxiety sensitivity in the context of a pandemic, as reported in previous literature, the research team selected factors associated with the COVID-19 pandemic. All the factors were probed as close-ended questions. The response options allowed various levels of choice. Generally, factors were divided into three categories.

The first category asked about the condition of the respondent's physical health. Participants were asked to indicate the following: (1) whether you have suffered from chronic disease in the 6 months prior to the pandemic (i.e., up to now, illness that has lasted for at least 6 months); (2) whether you have gone to see a doctor due to illness in the 3 months prior to the pandemic; and (3) whether you have gone to see a doctor due to illness during the period of the pandemic.

The second category dealt with the knowledge of COVID-19. Participants were asked the following: (1) Whether your family often talks about the COVID-19 pandemic. Answer options were: never, sometimes, or often. (2) How well do you understand the novel coronavirus and its outbreak (assessed by knowledge of its cause, transmission route, and preventive measures for COVID-19)? Answer options were: nothing, a little, or familiar. (3) What is your attitude toward taking self-protective measures (e.g., wearing a mask, hand washing)? Answer options were: actively, passively, or not taking any protective measures. (4) Whether you feel afraid or your heart beats fast when you hear things associated with COVID-19. Answer options were: never, sometimes, often, or always. (5) Whether family members have been involved in anti-COVID-19 efforts. Answer options were: yes or no. (6) Whether you perceive that the pandemic will have adverse impacts on yourself or your family in the future. Answer options were: yes or no. (7) Whether you think you might have COVID-19 at this time. Answer options were: yes or no. (8) Whether you have close contacts diagnosed with COVID-19. Answer options were: yes or no.

The last third and final category were about the routines of the participants. Participants were asked: (1) How much time do you spend on entertainment (e.g., playing games, listening to music, browsing the web, watching TV, etc.) per day? Answer options were <1, 1–2, or >2 h/day. (2) How much time do you spend on physical activity per day. Answer options were: <0.5, 0.5–1, or >1 h/day. (3) How much time do you spend studying per day? Answer options were: <1, 1–2, or >2 h/day.

#### The Chinese Version of the Childhood Anxiety Sensitivity Index

The Childhood Anxiety Sensitivity Index (CASI) is an 18-item self-reported Likert scale that can be used to assess anxiety sensitivity. It was developed by Silverman et al. (1999) on the basis of the Anxiety Sensitivity Index. The scale is rated on a 3-point scale ranging from 1 to 3 (“none” to “a lot”), and total scores range from 0 to 36, with higher scores indicating a higher level of anxiety sensitivity. Ren Fang (2008) demonstrated that the Chinese version of CASI has good reliability, validity, and strong internal consistency. The internal consistency for the present sample is 0.896, and the cutoff values are 9 for boys and 11 for girls, indicating anxiety sensitivity, respectively (Ren Fang, 2012). Although several hierarchical models have been proposed for the factor structure of CASI, in this study, a 3-factor model was administered because of its stability and consistency (Francis et al., 2019).

## Statistical Analysis

Categorical variables were presented as numbers and percentages, and continuous variables were presented as the mean ± SD. A two-sample independent *t-*test was used for comparisons between the two groups. One-way ANOVA was used for multigroup comparisons, and the least significant difference (LSD) method was used for pair comparisons. Multiple linear regression analysis was used to analyze factors influencing the CASI score. Binary logistic regression was used to analyze risk factors associated with high anxiety sensitivity. *p* < 0.05 was considered significant. All statistical tests were undertaken using SPSS Statistics software version 16.0 (IBM, Armonk, NY, USA).

## Results

A total of 1,141 questionnaires were retrieved in this study. Questionnaires with incomplete information and time spent of <90 s were deleted to ensure the reliability of data. In the final analysis, 1,091 (95.62%) questionnaires were included.

### Population Information

The general demographic data are shown in Table 1. Study participants comprised 593 females (54.4%) and 498 males (45.6%) with an average age of 13.27 ± 2.443 years. Among participants, 808 (74.1%) were of Han nationality, and 283 (25.9%) were others. In terms of education level, 524 (48.0%) respondents were enrolled in primary school, 296 (27.1%) were in secondary school, and 271 (24.8%) were in high school. Participants were from urban (19.6%), suburban (19.2%), and rural (61.1%) areas. Among respondents, 156 (14.3%) stated that their family members participated in anti-COVID-19 work. The average number of people living in the same home during the lockdown was 4.58 ± 1.258; among them, 948 (86.9%) were living with their parents, 398 (36.5%) were living with their grandparents, 726 (66.5%) were living with their siblings, and 48 (4.4%) were living with other people. Other respondent demographics and characteristics associated with the COVID-19 pandemic are presented in Table 1.

**Table 1:** Demographics and COVID-19-related characteristics of survey participants.

**Variable**		***N*** **= 1,091 (%)**
Gender	Female	593 (54.4)
	Male	498 (45.6)
Ethnicity	Han	808 (74.1)
	Others	283 (25.9)
Education level	Primary school	524 (48.0)
	Secondary school	296 (27.1)
	High school	271 (24.9)
Residence	Urban	214 (19.6)
	Suburban	210 (19.2)
	Rural	667 (61.2)
Living with parents during the lockdown	Yes	948 (86.9)
	No	143 (13.1)
Living with grandparents during the lockdown	Yes	398 (36.5)
	No	693 (63.5)
Living with siblings during the lockdown	Yes	726 (66.5)
	No	365 (33.5)
Living with others during the lockdown	Yes	48 (4.4)
	No	1,043 (95.6)
Suffered from chronic illness	Yes	32 (2.9)
in the previous 6 months	No	1,059 (97.1)
Suffered from illness	Yes	101 (9.3)
in the previous 3 months	No	990 (90.7)
Became ill and needed to go to hospital	Yes	197 (18.1)
during the lockdown	No	894 (81.9)
Discussion of COVID-19 pandemic	Never	74 (6.8)
among family members	Sometimes	404 (37.0)
	Often	613 (56.2)
Level of understanding of COVID-19	None	51 (4.7)
	A little	833 (76.4)
	Familiar	207 (18.9)
Attitude toward taking protective measures	Actively	738 (67.6)
	Passively	325 (29.8)
	Not taking any protective measures	28 (2.6)
Feeling afraid or having heart palpitations	Never	429 (39.3)
on hearing about COVID-19 pandemic	Sometimes	500 (45.8)
	Often	119 (10.9)
	Always	43 (4.0)
Family members involved	Yes	156 (14.3)
in anti-COVID-19 work	No	935 (85.7)
Perceived adverse impact of COVID-19	Yes	377 (34.6)
on self or family in future	No	714 (65.4)
Fear of infection	No	907 (83.1)
	Yes	184 (16.9)
Family members or friends	Yes	14 (1.3)
infected with COVID-19	No	1,077 (98.7)
Hours spent on entertainment	<1 h/day	247 (22.6)
	1–2 h/day	523 (47.9)
	>2 h/day	321 (29.5)
Hours spent on physical exercise	<1 h/day	323 (29.6)
	1–2 h/day	492 (45.1)
	>2 h/day	276 (25.3)
Hours spent on study	<1 h/day	84 (7.7)
	1–2 h/day	273 (25.0)
	>2 h/day	734 (67.3)

*COVID-19, coronavirus disease 2019*.

### Factors Associated With CASI Scores

The total CASI scores in this study ranged from 0 to 36, with an average score of 11.47 ± 6.631. Overall, 642 (58.8%) respondents reported anxiety sensitivity. Owing to sex differences in the threshold for anxiety sensitivity, the prevalence rates of anxiety sensitivity in female and male participants were 56.8 and 61.2%, respectively (**Table 5**). Additionally, as Table 2 shows, an in-depth analysis of three dimensions of anxiety sensitivity found that the levels of physical concerns, mental concerns, and social concerns differed significantly between respondents with or without anxiety sensitivity.

**Table 2:** CASI scale scores ($\bar{x}$ ± *SD*).

**Variable**	**All respondents (*N* = 1,091)**	**Respondents with anxiety sensitivity (*N* = 642)**	**Respondents without anxiety sensitivity (*N* = 449)**	***t*** **value**	***p***
Physical concerns	7.23 ± 4.773	10.33 ± 3.480	2.81 ± 2.186	43.758	<0.001
Mental concerns	1.45 ± 1.464	2.19 ± 1.405	0.39 ± 0.699	27.854	<0.001
Social concerns	2.79 ± 1.346	3.34 ± 1.112	1.99 ± 1.251	18.729	<0.001
CASI score	11.47 ± 6.631	15.86 ± 4.683	5.19 ± 2.921	46.249	<0.001

*CASI, Childhood Anxiety Sensitivity Index*.

One-way ANOVA and the *t*-test were used to analyze factors influencing CASI scores. The results are shown in Table 3. Several factors were significantly related to CASI scores: gender, ethnicity, educational level, physical condition during the lockdown, discussion about COVID-19 within the family, knowledge about COVID-19, attitude toward taking protective measures, feeling afraid or experiencing rapid heartbeat or palpitations on hearing about COVID-19, perceiving that COVID-19 had adverse impacts on self or family, family members being involved in anti-epidemic work, and fear of infection. Further analysis by the LSD method found that the scores of high school students were significantly higher than those of secondary school students (*p* = 0.045), and those of secondary school students were significantly higher than those of primary school students (*p* = 0.002). The scores of those whose family members discussed the pandemic of COVID-19 sometimes (*p* = 0.001) or often (*p* < 0.001) were significantly higher than those whose family members did not. The scores of those who thought they were familiar with (*p* = 0.002) or knew a little (*p* < 0.001) about COVID-19 were significantly higher than those who did not (*p* < 0.001); the scores of those who took protective measures actively (*p* = 0.015) or passively (*p* = 0.016) were significantly higher than those who did not. Lastly, the scores of those who felt afraid and alarmed to the point of experiencing rapid heartbeats or palpitations when hearing things related to the epidemic were significantly higher than those who did not have these responses (*p* < 0.001).

**Table 3:** CASI scores by variable.

**Variables**		**CASI ($\bar{x}$ ± *SD*)**	***t/F***	***p***
Gender	Female	12.00 ± 6.449	2.928^*^	0.003
	Male	10.83 ± 6.793		
Ethnicity	Han	11.86 ± 6.697	3.360^*^	0.001
	Others	10.33 ± 6.314		
Education level	Primary school	10.42 ± 6.248	14.997	<0.001
	Secondary school	11.91 ± 6.942		<0.000^a^
	High school	13.01 ± 6.668		0.045^b^
Residence	Urban	10.95 ± 6.465	1.043	0.353
	Suburban	11.87 ± 6.521		
	Rural	11.50 ± 6.717		
Living with parents during the lockdown	No	10.85 ± 7.354	−1.098^*^	0.274
	Yes	11.56 ± 6.514		
Living with grandparents during the lockdown	No	11.18 ± 6.739	−1.902^*^	0.057
	Yes	11.97 ± 6.415		
Living with siblings during the lockdown	No	11.18 ± 6.820	−0.999^*^	0.318
	Yes	11.61 ± 6.534		
Living with others during the lockdown	No	11.45 ± 6.607	−0.436^*^	0.663
	Yes	11.88 ± 7.192		
Suffered from chronic illness in the previous 6 months	Yes	13.72 ± 7.587	1.953^*^	0.051
	No	11.40 ± 6.592		
Suffered from illness in the previous 3 months	Yes	12.41 ± 6.771	1.495^*^	0.135
	No	11.37 ± 6.612		
Became ill and needed to go to hospital during the lockdown	Yes	12.40 ± 6.444	2.189^*^	0.029
	No	11.26 ± 6.657		
Discussion of COVID-19 pandemic among family members	Never	8.42 ± 7.038	9.719	<0.001
	Sometimes	11.29 ± 6.521		0.001^c^
	Often	11.95 ± 6.559		<0.001^c^
Level of understanding of COVID-19	Nothing	7.80 ± 7.733	9.652	<0.001
	A little	11.81 ± 6.404		0.000^d^
	Familiar	10.97 ± 6.959		0.002^d^
Attitude toward taking protective measures	Not taking any protective measures	8.43 ± 8.871	3.051	0.048
	Passively	11.61 ± 6.662		0.016^e^
	Actively	11.52 ± 6.501		0.015^e^
Feeling afraid or having heart palpitations on hearing about COVID-19 pandemic	Never	9.09 ± 6.320	33.653	<0.001
	Sometimes	12.81 ± 6.089		<0.001^f^
	Often	13.51 ± 7.551		<0.001^f^
	Always	13.88 ± 6.036		<0.001^f^
Family members involved in anti-COVID-19 work	Yes	12.71 ± 6.160	2.526^*^	0.012
	No	11.26 ± 6.687		
Perceived adverse impact of COVID-19 on self or family in future	Yes	13.26 ± 6.484	6.628^*^	<0.001
	No	10.52 ± 6.514		
Fear of infection	Yes	13.33 ± 7.549	−3.768^*^	<0.001
	No	11.09 ± 6.367		
Family members or friends infected with COVID-19	Yes	13.07 ± 6.911	0.911^*^	0.362
	No	11.45 ± 6.628		
Hours spent on entertainment	<1 h/day	11.78 ± 7.035	0.485	0.616
	1–2 h/day	11.47 ± 6.406		
	>2 h/day	11.22 ± 6.681		
Hours spent on physical exercise	<1 h/day	11.96 ± 6.691	2.042	0.130
	1–2 h/day	11.48 ± 6.680		
	>2 h/day	10.87 ± 6.445		
Hours spent on study	<1 h/day	12.56 ± 6.541	1.407	0.245
	1–2 h/day	11.18 ± 6.594		
	>2 h/day	11.45 ± 6.651		

* *t test*.

a *Compared to those of secondary school students*.

b *Compared to those of high school students*.

c *Compared to those whose family members never discussed the pandemic of COVID-19*.

d *Compared to those who thought they knew nothing about COVID-19*.

e *Compared to those who did not take any protective measures*.

f *Compared to those who never felt afraid and alarmed to the point of experiencing rapid heartbeats or palpitations when hearing things related to the epidemic*.

*CASI, Childhood Anxiety Sensitivity Index*.

In addition, we conducted multiple linear regression analysis by a variable with the aforementioned significant factors, age, and number of people in the same home during the lockdown in order to identify the significant factors correlated with the level of anxiety sensitivity. Finally, we constructed a multiple linear regression model of anxiety sensitivity scores from the factors obtained (Table 4), including gender, school grade level, seeking medical help because of illness during the lockdown, feeling afraid, or experiencing rapid heartbeat on hearing things related to COVID-19, family members participating in anti-COVID-19 work, perceiving that COVID-19 would have an adverse impact on self or family, and fear of infection.

**Table 4:** Factors related to respondents' level of anxiety sensitivity during the COVID-19 outbreak.

**Model**	**Unstandardized coefficients'**		**Standardized coefficients**	***t-*** **test score**	***p value***	**95% CI**
	***B***	***SE***				
(Constant)	7.749	2.647		2.927	0.003	2.554~12.943
Gender	1.224	0.377	0.092	3.244	0.001	0.484~1.965
Age	−0.161	0.518	−0.059	−0.987	0.324	−0.482~0.159
Ethnicity	0.231	0.149	0.015	0.446	0.656	−0.786~1.248
Education	1.968	0.489	0.244	3.753	0.000	0.939~2.997
Number of people living together during the lockdown	0.050	0.333	0.009	0.332	0.740	−0.243~0.342
Became ill and needed to go to hospital during the lockdown	−1.047	0.423	−0.061	−2.140	0.033	−2.006~−0.087
Discussion of COVID-19 in family	0.240	0.379	0.023	0.723	0.470	−0.412~0.893
Level of understanding of COVID-19	−0.119	0.253	−0.008	−0.281	0.779	−0.948~0.711
Attitude toward taking protective measures	−0.514	0.541	−0.039	−1.357	0.175	−1.258~0.229
Feeling afraid or having heart palpitations on hearing about COVID-19	1.961	0.416	0.233	7.738	0.000	1.464~2.458
Family members involved in anti- COVID-19 work	−1.188	0.505	−0.063	−2.197	0.028	−2.249~−0.127
Perceived adverse impact of COVID-19 on self or family in future	−1.442	0.163	−0.103	−3.465	0.001	−2.258~−0.625
Fear of infection	2.091	0.524	0.118	4.141	0.000	1.100~3.081

*F = 14.329; p < 0.001; R^2^ = 0.147; adjusted R^2^ = 0.137. Stepwise selection procedure was admitted to select the model as well as variables including age, number of people living together during lockdown, and significant factors in the level of anxiety sensitivity (total CASI scores) listed in Table 4*.

### Risk Factors for Anxiety Sensitivity in Children and Adolescents

We performed a binary logistic regression analysis to identify risk factors for anxiety sensitivity in children and adolescents from northwest China. As Table 5 shows, there were several risk factors for anxiety sensitivity in children and adolescents during lockdown: learning stage in secondary school (OR, 1.743; 95% CI [1.274–2.384]) or high school (OR, 2.151; 95% CI [1.544–2.997]); becoming ill and needing to go to hospital during the lockdown (OR, 1.462; 95% CI [1.038–2.059]); being afraid of hearing things related to COVID-19 either sometimes (OR, 2.900; 95% CI [2.187–3.846]), often (OR, 2.522; 95% CI [1.595–3.988]), or always (OR, 4.061; 95% CI [1.945–8.480]); believing that COVID-19 would have an adverse impact on self or family (OR, 1.513; 95% CI [1.135–2.017]); and fear of infection (OR, 1.703; 95% CI [1.187–2.444]).

**Table 5:** Factors related to anxiety sensitivity in children and adolescents during the COVID-19 lockdown.

**Variable**	**Frequency *n* (%) of** **anxiety sensitivity** **(*N* = 642)**	***p value***	**Odds ratio (95%CI)**
**Gender**			
Female	337 (56.8%)		1
Male	305 (61.2%)	0.070	1.272 (0.981–1.650)
**Education level**			
Primary school	272 (51.9%)		1
Secondary school	188 (63.5%)	0.001	1.743 (1.274–2.384)
High school	182 (67.2%)	<0.001	2.151 (1.544–2.997)
**Became ill and needed to go to hospital during the lockdown**			
No	513 (57.4%)		1
Yes	129 (65.5%)	0.030	1.462 (1.038–2.059)
**Feeling afraid or having heart palpitations on hearing about COVID-19**			
Never	184 (42.9%)		1
Sometimes	346 (69.2%)	<0.001	2.900 (2.187–3.846)
Often	80 (67.2%)	<0.001	2.522 (1.595–3.988)
Always	32 (74.4%)	<0.001	4.061 (1.945–8.480)
**Family members involved in anti- COVID-19 work**			
No	539 (57.6%)		1
Yes	103 (66.0%)	0.099	1.378 (0.941–2.018)
**Perceived adverse impact of COVID-19 on self or family in future**			
No	377 (52.8%)		1
Yes	265 (70.3%)	0.005	1.513 (1.135–2.017)
**Fear of infection**			
No	514 (70.3%)		1
Yes	128 (52.8%)	0.004	1.703 (1.187–2.444)

*Forward stepwise selection procedure was conducted to select the model from variables listed in Table 4 that had significant differences in levels of anxiety sensitivity*.

## Discussion

This is one of few studies, to our knowledge, that describes the psychological condition of children and adolescents from the northwest China during the pandemic. We found that the level of anxiety sensitivity became dramatically elevated during the pandemic. We also revealed several possible risk factors associated with high anxiety sensitivity: studying in secondary or high school, becoming ill, feeling afraid or having heart palpitations on hearing about the COVID-19 pandemic, thinking that COVID-19 would have an adverse impact on self or family in the future, and fear of infection. Together, these results will help us to better understand the mental health conditions of children and adolescents when faced with current or future emerging infectious disease outbreaks and epidemics. Thus, we will be able to provide scientific guidance to formulate targeted policies to prevent such mental illness and intervene when it occurs.

Initially, our results showed that the level of anxiety sensitivity in children and adolescents from northwest China during the pandemic increased significantly, exceeding that of children and adolescents assessed prior to the outbreak of COVID-19 (Ren Fang, 2012). Due to the lack of data on the anxiety sensitivity of children and adolescents in other parts of China during the pandemic, it is not possible to compare the levels of anxiety sensitivity of children and adolescents in northwest China with those from other parts of China. In our sample, 58.8% of participants met the screening criteria for anxiety sensitivity. In related research, Tang et al. (2021) reported on the prevalence of depressive symptoms (19.7%) and anxiety symptoms (24.9%) in children and adolescents from Shanghai during the pandemic, and Xie et al. (2020) reported on the prevalence of depressive symptoms (26.5%) and anxiety symptoms (19.6%) in children and adolescents in Wuhan. Although the rate of abnormal psychological status among children and adolescents varied in the different studies, these findings consistently suggest that the COVID-19 pandemic has had an adverse impact on the psychological status of children and adolescents.

As is well-known, the physical disease can influence psychological conditions. However, the most interesting and concerning findings of the present study are that becoming ill and needing medical treatment during the lockdown was a risk factor for anxiety sensitivity, whereas having the chronic disease in the preceding 6 months or experiencing illness and seeking medical advice in the past 3 months was not a risk factor. This difference might be due to the variety of clinical symptoms of COVID-19 (Huang et al., 2020). Also, it is difficult for children and adolescents to distinguish the symptoms of the general disease from those of COVID-19, and they may have guessed that they were infected with COVID-19, thus elevating their levels of anxiety sensitivity. Those with chronic diseases or experience seeking medical advice previously, by contrast, knew their health conditions well and were less likely to make false assumptions and guesses.

Similar to the findings of Zhou et al. (2020b), our study revealed that the higher the school grade level, the higher the CASI score. This correlation may be due to the fact that middle school students attach more importance to their academic achievements and interpersonal communication (Wang et al., 2007). Moreover, as school age increases, students' academic stress significantly increases, and interpersonal relationships become more complicated. After the outbreak of COVID-19, lockdown measures and postponement of the spring semester disturbed learning schedules and daily life (Fang et al., 2019, 2021c; Wu et al., 2022b). Although students could study and communicate online, poor learning efficiency and restrictions on communication with peers may have increased their anxiety sensitivity.

The COVID-19 pandemic is the most serious public health event these children and adolescents have experienced. In this survey, 62.4% of the respondents felt afraid and experienced a rapid heartbeat when they heard information about COVID-19. Further analysis found that concerns about the adverse impacts of COVID-19 on themselves and their families in the future, fears of being infected with COVID-19, and fears upon hearing information about the pandemic were all risk factors for anxiety sensitivity. On the one hand, this might be related to their young age and lack of mental resilience in response to adversity (Liu Wen and Lin, 2019). On the other hand, a virus that is highly contagious has a high rate of mortality, has no specific treatment, and has increasing numbers of confirmed cases and deaths might have aggravated their fears and anxiety sensitivity. Meanwhile, in order to control the spread of disease, governments implementing strict lockdowns might also have disturbed parents' careers and family economics (Chen et al., 2022; Fang et al., 2022), while postponement of school re-openings might have interfered with children's schoolwork, resulting in enhanced anxiety sensitivity among children and adolescents.

Because the pandemic was ongoing during the investigation period, the study had to take a convenience sampling approach and be conducted online; thus, the sample size is relatively small, which limits the applicability and generalizability of the results. Also, because of the nature of a cross-sectional study, the ability to establish causal relationships between risk factors and anxiety sensitivity was limited. Therefore, longitudinal follow-up studies should be conducted that expand sample sources and investigate the respondents face to face. This will improve the study design and increase the applicability and generalizability of the results.

## Conclusion

During the COVID-19 pandemic and home quarantine, children and adolescents from northwest China experienced elevated levels of anxiety sensitivity. Therefore, the whole society should be aware of the negative impact of the pandemic on the mental health of children and adolescents and develop timely and effective interventions to prevent and intervene during pandemics so as to avoid more severe and disabling consequences. Specifically, parents should pay more attention to the physical health of their children during lockdowns and help them to seek medical help as soon as symptoms appear. Doctors should give them professional advice and allay their doubts and concerns about COVID-19, thereby reducing their anxiety sensitivity. The media should report information related to the pandemic accurately to avoid excessive exaggeration of its seriousness. At the same time, because the pandemic is dangerous, it is imperative to take strict control measures that interfere with people's daily routines. Schools and parents should encourage students to view the pandemic from a long-term perspective with a positive, optimistic aspect. In this way, they will help children to accept and adapt to lockdown measures with heartfelt understanding so as to relieve their anxiety.

## Data Availability Statement

The raw data supporting the conclusions of this article will be made available by the authors, without undue reservation.

## Ethics Statement

The studies involving human participants were reviewed and approved by the Second Affiliated Hospital of Xi'an Jiaotong University. Written informed consent from the participants' legal guardian/next of kin was not required to participate in this study in accordance with the national legislation and the institutional requirements.

## Author Contributions

QJ, WM, and YZ designed the questionnaire, organized the survey, and carried out the literature searches and manuscript preparation. HW, YG, and JH assisted with data acquisition and analyses. BZ and JL carried out manuscript editing. WH and SL undertook a manuscript review. All authors approved the final version of the manuscript.

## Funding

This work was supported by the Foundation of the Second Affiliated Hospital of Xi'an Jiaotong University [Grant No. YJ (QN)201808] and the Project of Shaanxi Province to Improve Public Science Quality (Grant No. 2021PSL93).

## Conflict of Interest

The authors declare that the research was conducted in the absence of any commercial or financial relationships that could be construed as a potential conflict of interest.

## Publisher's Note

All claims expressed in this article are solely those of the authors and do not necessarily represent those of their affiliated organizations, or those of the publisher, the editors and the reviewers. Any product that may be evaluated in this article, or claim that may be made by its manufacturer, is not guaranteed or endorsed by the publisher.
